# The International Veterinary Congress

**Published:** 1884-01

**Authors:** 


### THE INTERNATIONAL VETERINARY CONGRESS.

A new epoch in the history of veterinary medicine has been marked by the assemblage of the Congress of Veterinary Surgeons, which occurred in Brussels on September 10th at the Palais des Académies.

The object of the meeting, the interesting material there proposed for discussion and the results already obtained impress us with the conviction that this Congress will have a far-reaching influence in all matters that tend to the progress and welfare of the veterinary profession.

This is the fourth and most important of a series of meetings of the Congress, extending over a period of twenty years, at which international laws relating to cattle plagues and other professional matters were discussed.

The United States of America and all countries of Europe, with the exception of Russia, Italy, Spain and Portugal, were represented, the delegates from this country being Professors Law, Liautard and Robertson.

The following were the subjects presented:

*The Organization of Veterinary Service.*—It was resolved that such a service should employ the services of as many veterinary surgeons as possible in the inspection of fairs and markets, abattoirs and slaughter houses, etc., and should be an international organization, having special relation to the oppression and prevention of contagious diseases.

*Contagious Pleuro-pneumonia.*—The special characters of the disease were discussed, its origin, spontaneous, or by infection, preventive measures, isolation, disinfection and inoculation. Difference of opinion existed among the members as to the origin of the disease, but the majority were in favor of regarding it as infectious, and, in the conclusions submitted, inoculation as a prophylactic measure is recommended.

In the question of inoculation we are pleased to note that the Congress agrees with the opinions expressed thereon in our last number in an editorial note on the action of the N. J. State Board of Health in this matter.

Professor Law, in his remarks on the subject, said that inoculation in pleuro-pneumonia had the same value as vaccination in variola, but that to completely destroy a center of infection slaughter was a more efficacious and radical measure. Mr. Fleming advised inoculation in countries notably infected with the disease, and in large herds where slaughtering would be ruinous.

It was stated that researches so far made have failed to discover the microbe of the disease.

*Education in Veterinary Medicine.*—Candidates for admission to the study of veterinary medicine must have terminated the studies of the preliminary branches of education. At least four years must have been devoted to special studies by the candidates for graduation.

During the discussion an objection was raised against the requirements for the study of veterinary medicine being the same as human medicine, the former having for its object the conservation of things whose value may be estimated by a money standard, which is not true of the latter, as man has a value that cannot be thus defined. This opinion was assented to with the qualification, however, that the veterinarian is obliged to inform himself in reference to hygienic questions, and thus becomes the equal of the physician. In support of the latter view we might enumerate the many benefits which human medicine has derived, in the past, from the labors of investigators in comparative medicine and call attention to the endeavors being made at the present moment with every prospect of success, to obtain a means of preventing that terrible scourge to humanity—scarlatina.

*Tuberculosis.*—Three important questions relative to this subject were discussed, viz :

First.—What is the influence of heredity upon its propagation?

Second.—What is the influence of contagion?

Third.—What are the preventive measures to be employed against the bad influence that may be produced by the utilization of the meat and milk of animals affected with the disease?

The first two questions were answered by the conclusion arrived at that tubercle is propagated by heredity and by contagion, as is proved by the cases quoted in support of this opinion.

In the pages of the JOURNAL the second of these questions has been discussed at different times, the writers all agreeing in their views as to the infectious nature of tuberculosis, basing their convictions especially on the discovery of the "bacillus" of tubercle.

No reference was made in the Congress to the lately described disease, Actinomykosis, which is, no doubt, in many cases mistaken for the disease under consideration.

The transmissability of the disease being admitted, the importance of the third question is evident, as is also the necessity of strict sanitary regulations in regard to the consumption of animal food.

Mr. Lydtin, in opening the discussion, said, "phthisis is so common a disease that it deserves before any other ailment the name of *universal panzootic.*

"This disease not only touches the preservation of our cattle, also the health of man. If we succeed in solving the question, we shall have reached a noble object, that of protecting at the same time the prosperity and health of the public. Such results will seem to us our just reward in the esteem, consideration and gratitude of the whole world."

Among the resolutions proposed for the adoption of the Congress were the following:

"Tuberculosis is a malady which is transmissable by heredity and by contagion. It is a disease which ought to be combatted by measures of sanitary police.

Every stockholder should be compelled to declare the existence of tuberculosis among his animals to the police authorities, and even to give notice when he observes any symptoms which lead him to suspect the existence of the disorder. It should also be cumpulsory to put the suspected animals in such a position as to avoid the risk of the extension of the disease to other animals.

This obligation is to be made to apply to a person having charge of animals in transit, and also to the owner of a stable or pastures in which or on which animals are kept. The same obligation to give notice of the tuberculosis is to be laid on all veterinary surgeons or persons concerned in treatment of the diseases of domestic animals, also on all whose business it is to destroy or utilize, or in any way manipulate, the carcasses of animals. The existence of the disease and its locality is to be made public, and even the herd in which it has appeared is to be named. Diseased and suspected animals should be isolated and their slaughter ordered. If the number of suspected animals is very large it may suffice to advise the feeder to send them to the abattoir as soon as possible.

Herds and places infected are to be under the observation of the police for a year after the occurrenc of the last case of disease, and the sale of suspected animals is only to be permitted when they are destined to immediate slaughter. Disinfection is to be insisted on, and not till after it has been carried out should the police regulations, applying to the places in which diseased animals have been kept, be relaxed.

Meat from tuberculous animals is not to be sold for consumption unless it is certain that the disease was in the incipient stage, and that the meat presents the appearance of being first-rate in quality.

The inspection of animals affected with tuberculosis is to be performed by a veterinary surgeon *who is to be the sole judge as to the fitness or otherwise of the meat for human food.*

Milk of diseased animals is not to be used for food of man or animals; and the milk of animals which are suspected of contaminatjon is not to be used until after it has been boiled.

Compensation to owners of diseased animals for losses sustained from the operation of the above police measures is to be provided by the State; and, as a means of raising the necessary funds, it is proposed to adopt a system of compulsory insurance, every owner of cattle being compelled to subscribe a certain sum in proportion to the size of his herd."

Want of time prevented the Congress from discussing these propositions and therefore no resolutions were passed.

The editor of *La Clinica Veterinario*, alluding to the fact that Italy was not represented in the Congress throws the blame on the government and the official veterinarians of that country, and bewails the scepticism in regard to the profession there and the backward state generally of veterinary matters.

